# Advanced Glycation End Products Inhibit the Proliferation of Human Umbilical Vein Endothelial Cells by Inhibiting Cathepsin D

**DOI:** 10.3390/ijms18020436

**Published:** 2017-02-17

**Authors:** Yuan Li, Ye Chang, Ning Ye, Dongxue Dai, Yintao Chen, Naijin Zhang, Guozhe Sun, Yingxian Sun

**Affiliations:** 1 Department of Cardiology, The People’s Hospital of Liaoning Province, Shenyang 110001, China; xi.aohan1989@163.com; 2 Department of Cardiology, The People’s Hospital of China Medical University, Shenyang 110001, China; 3 Department of Cardiology, The First Hospital of China Medical University, Shenyang 110001, China; chang.ye@stu.xjtu.edu.cn (Ye.C.); yening_cmu@163.com (N.Y.); 18240409506@163.com (D.D.); pinkrainsky@foxmail.com (Yi.C.); zhangnj1989@126.com (N.Z.); gzhsun66@163.com (G.S.)

**Keywords:** advanced glycation end products (AGEs), human umbilical vein endothelial cells (HUVECs), proliferation, migration, cathepsin D (CTSD), autophagy

## Abstract

We aimed to investigate the effect of advanced glycation end products (AGEs) on the proliferation and migration ability of human umbilical vein endothelial cells (HUVECs). Cell proliferation was detected by methyl thiazolyl tetrazolium (MTT) assay, real-time cell analyzer and 5-Ethynyl-2′-deoxyuridine (EdU) staining. Cell migration was detected by wound-healing and transwell assay. AGEs significantly inhibited the proliferation and migration of HUVECs in a time-and dose-dependent way. Western blotting revealed that AGEs dramatically increased the expression of microtubule-associated protein 1 light chain 3 (LC3) II/I and p62. Immunofluorescence of p62 and acridine orange staining revealed that AGEs significantly increased the expression of p62 and the accumulation of autophagic vacuoles, respectively. Chloroquine (CQ) could further promote the expression of LC3 II/I and p62, increase the accumulation of autophagic vacuoles and promote cell injury induced by AGEs. In addition, AGEs reduced cathepsin D (CTSD) expression in a time-dependent way. Overexpression of wild-type CTSD significantly decreased the ratio of LC 3 II/I as well as p62 accumulation induced by AGEs, but overexpression of catalytically inactive mutant CTSD had no such effects. Only overexpression of wild-type CTSD could restore the proliferation of HUVECs inhibited by AGEs. However, overexpression of both wild-type CTSD and catalytically inactive mutant CTSD could promote the migration of HUVECs inhibited by AGEs. Collectively, our study found that AGEs inhibited the proliferation and migration in HUVECs and promoted autophagic flux, which in turn played a protective role against AGEs-induced cell injury. CTSD, in need of its catalytic activity, may promote proliferation in AGEs-treated HUVECs independent of the autophagy-lysosome pathway. Meanwhile, CTSD could improve the migration of AGEs-treated HUVECs regardless of its enzymatic activity.

## 1. Introduction

Advanced glycation end-products (AGEs) are modified proteins or lipids resulting from non-enzymatic glycosylation reactions [1,2]. Accumulating evidence indicates that AGEs play a crucial role in the process of atherosclerosis (AS) [3,4]. In the pathophysiological process of AS, the interaction between AGEs and endothelial cells has been reported to be an important event [3,4]. AGEs are mainly located in the vessel wall and lead to the dysfunction of vascular endothelial cells characterized by the imbalance between proliferation and death of endothelial cells, increased endothelial permeability, and finally the development of atherosclerosis [5,6]. Increasing evidence has reported that AGEs could induce the apoptosis in HUVECs via multiple signaling pathways such as the receptor of advanced glycation end products (RAGE)/nuclear factor-κB (NF-κB) pathway [7,8], nitric oxide (NO)/cyclic guanosine monophosphate (cGMP) signaling [9], targeting insulin-like growth factor 1 receptor [10], and the mitochondrion-cytochrome c-caspase protease pathway [11]. However, whether AGEs can promote or inhibit the proliferation and migration of human umbilical vein endothelial cells (HUVECs) is still a matter of debate [12,13,14,15].

Autophagy is an evolutionarily conserved process whereby unnecessary or dysfunctional cytoplasmic proteins and cellular organelles can be degraded in lysosomes for amino acid and energy recycling [16,17]. As a mechanism of self-digestion, autophagy can provide an alternative energy source and serve an adaptive role to protect cells against extreme stress conditions such as starvation [16,17]. The autophagy process involves many genes and organelles. It is well accepted that misfolded proteins and cell debris are sequestered within autophagosome, which then fuse with lysosomes to form autolysosomes [4]. The autophagic–lysosomal pathway plays an important role in the degradation of cellular structures [4]. Previous studies demonstrated that autophagy could reduce cell injury and proliferation in HUVECs induced by oxidized low density lipoprotein (ox-LDL) [18], glycated collagen I [19], and AGEs [3]. However, the mechanism by which AGEs induce the dysfunction of HUVECs and the role of autophagy in the process is yet to be investigated.

The lysosomal proteases cathepsins are important mediators of autophagy. Cathepsin D (CTSD), an aspartyl protease in lysosomes, participates directly in autophagy and is responsible for the degradation of cellular structures [20]. Our previous study indicated that CTSD overexpression could suppress AGE-induced proliferation of rat vascular smooth muscle cells (VSMCs) while CTSD overexpression had no effects on the migration of VSMCs induced by AGEs [21]. However, there are rare studies about the effects of autophagy on the AGE-induced endothelial injury.

Thus, this study aimed to elucidate the role of autophagy in the proliferation and migration in HUVECs mediated by AGEs and its underlying mechanisms.

## 2. Results

### 2.1. Advanced Glycation end-Products (AGEs) Inhibited the Proliferation of Human Umbilical Vein Endothelial Cells (HUVECs)

**Figure 1:**
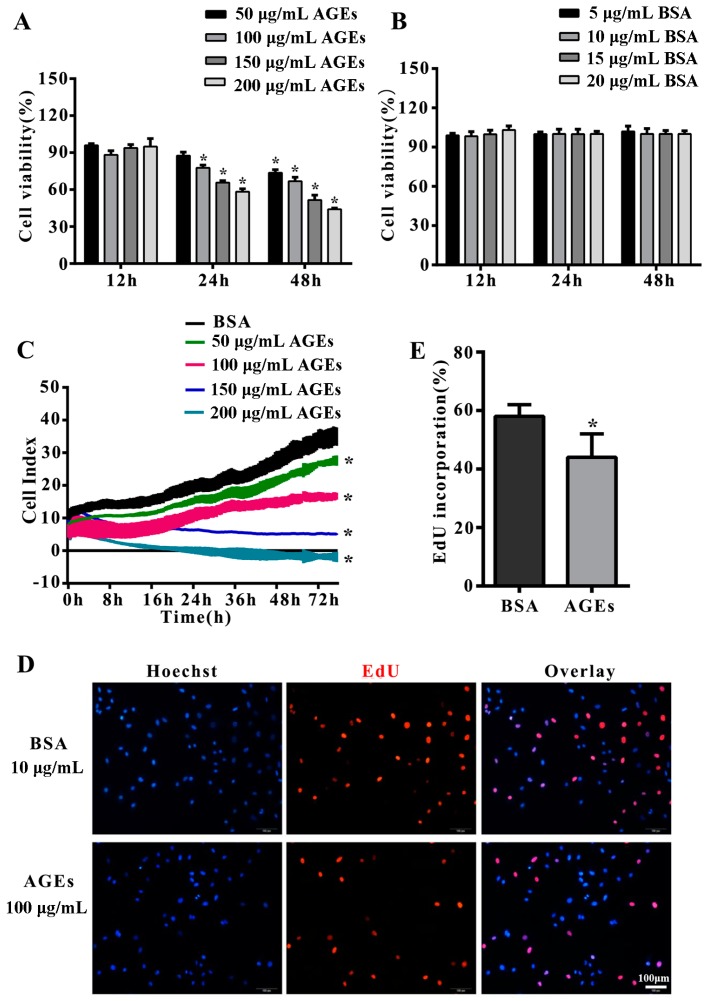
Advanced glycation end products (AGEs) inhibited the proliferation of human umbilical vein endothelial cells (HUVECs). Cells were incubated with different concentrations of AGEs (50, 100, 150 and 200 µg/mL) or different concentrations of bovine serum albumin (BSA) (5, 10, 15 and 20 µg/mL) as control for different times (12, 24 and 48 h). (**A**,**B**) Cell proliferation was detected by Methyl thiazolyl tetrazolium (MTT) assay (* *p* < 0.05 vs. BSA group at corresponding concentration and the same time-point); (**C**) Cell proliferation was monitored by a real-time cell analyzer (RTCA) dynamically. Cell index was recorded automatically every 15 min intervals and monitored continuously for 48 h (* *p* < 0.05 vs. BSA group); (**D**) Cell proliferation was also detected by 5-Ethynyl-2′-deoxyuridine (EdU) staining assay, in which the red dots represented the population of newborn cells. Hoechst 33342 staining (blue dots) was used to label cell nuclei; (**E**) Three microscopic fields were randomly selected. EdU incorporation (%) was quantified by Image J 1.47, which was calculated by the formula: EdU incorporation = cells stained by red/(cells stained by red + cells stained by blue) × 100% (* *p* < 0.05 vs. BSA group). All data were obtained from reproducible experiments (*n* = 3).

To study the effect of AGEs on cells proliferation, HUVECs were incubated with different concentrations of AGEs (50, 100, 150 and 200 µg/mL) or different concentrations of bovine serum albumin (BSA) (5, 10, 15 and 20 µg/mL) as control for different times (12, 24 and 48 h). Methyl thiazolyl tetrazolium (MTT) assay demonstrated that AGE treatment could dramatically decrease the proliferation of HUVECs in a time- and concentration-dependent manner (Figure 1A,B). This was confirmed by the results of the real-time cell analyzer (RTCA) which could monitor cell proliferation continuously and dynamically (Figure 1C). Based on these results, we treated HUVECs with 100 µg/mL AGEs for 24 h in subsequent experiments. 5-Ethynyl-2′-deoxyuridine (EdU) incorporation assay indicated AGEs could significantly inhibit DNA synthesis in HUVECs (Figure 1D,E).

### 2.2. AGEs Inhibited the Migration of HUVECs

**Figure 2:**
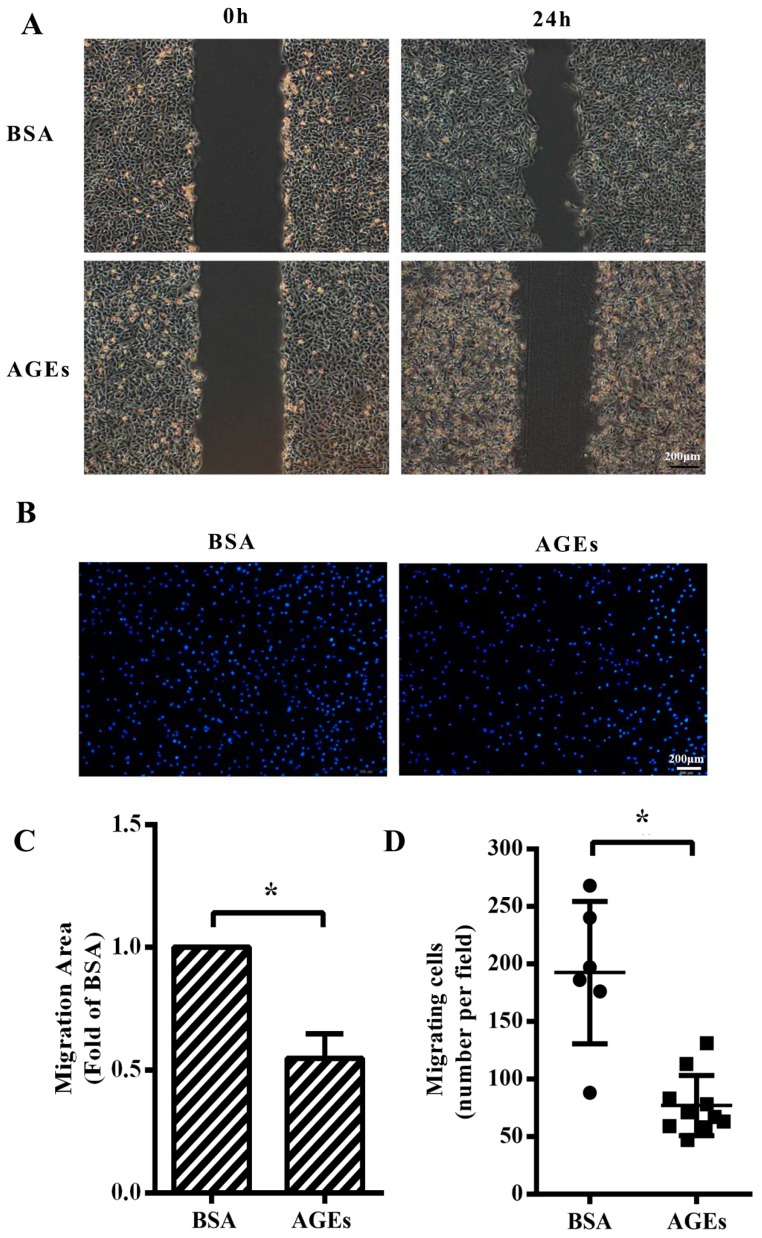
AGEs inhibited the migration of HUVECs. Cells were treated with 100 µg/mL AGEs or 10 µg/mL BSA as control, and incubated with serum-free Dulbecco’s modified Eagle medium (DMEM) for 24 h to inhibit cell proliferation. (**A**) Cell migration was detected by wound-healing assay; (**B**) Migrating cells were detected by Hoechst 33342 staining in the transwell assay. Blue dots represented nuclei; (**C**) Migration area in wound healing assay was quantified using Image J 1.47. Three microscopic fields were randomly selected; (**D**) Migrating cells in transwell assay were calculated using Image J 1.47. Three microscopic fields were randomly selected. All data were obtained from reproducible experiments (*n* = 3). * *p* < 0.05 vs. BSA group.

We next aimed to assess whether AGEs (100 µg/mL) or BSA (10 µg/mL) had any influence on the migration of HUVECs. The results from wound-healing assay manifested that cellular migration capacity was impaired in AGEs group compared with BSA-group (Figure 2A,C). Consistently, transwell migration assay also showed that the numbers of migrating cells were significantly lower in the AGEs-treated group than those in the BSA group (Figure 2B,D).

### 2.3. AGEs Promoted Autophagic Flux in HUVECs

**Figure 3:**
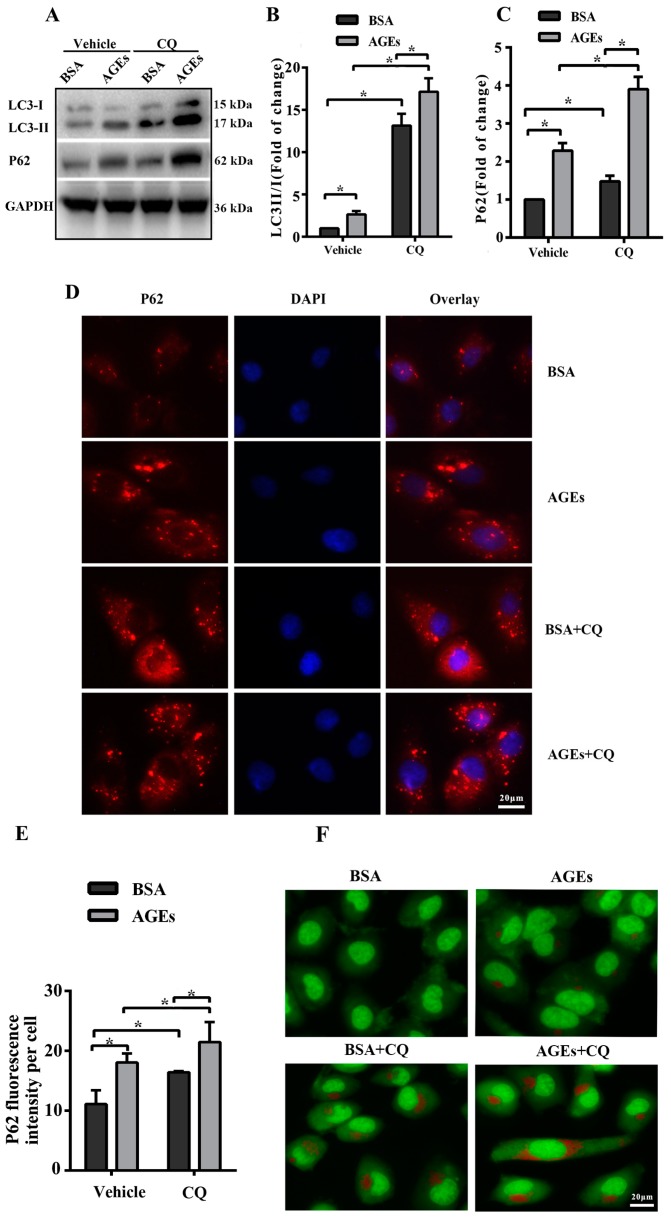

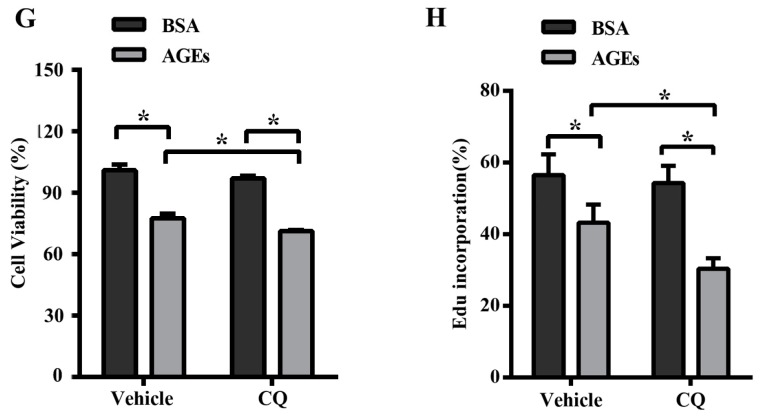
AGEs promoted autophagic flux in HUVECs. Cells were incubated with 10 µM chloroquine (CQ) for 30 min before exposure to 100 µg/mL AGEs and 10 µg/mL BSA for 24 h. (**A**) The protein expression of microtubule-associated protein 1 light chain 3 (LC3)-II/I and p62 were detected using western blotting, and glyceraldehyde 3-phosphate dehydrogenase (GAPDH) served as a control; (**B**,**C**) The band intensity was quantified using Image J 1.47, normalized by GAPDH; (**D**) After treatment, immunofluorescence was conducted. Cells were stained for p62 (red) and the nuclear marker 4′,6-diamidino-2-phenylindole (DAPI) (blue); (**E**) The fluorescence intensity of p62 was quantified using Image J 1.47. Three microscopic fields were randomly selected; (**F**) Autophagic vacuoles were detected by acridine orange (AO) staining. Red dots represented autophagic vacuoles; (**G**) Cell viability was detected by MTT assay; (**H**) Cell proliferation was detected by EdU assay. Three microscopic fields were randomly selected. EdU incorporation (%) was quantified by Image J 1.47, which was calculated by the formula: EdU incorporation = cells stained by red/(cells stained by red + cells stained by blue) × 100%. All data were obtained from reproducible experiments (*n* = 3). * *p* < 0.05 compared with respective control.

The dynamic change of autophagy contains two aspects: the formation and degradation of autophagosome. When autophagy is induced, microtubule-associated protein 1 light chain 3 (LC3) will transform from the cytoplasmic form (LC3-I) to a membrane-associated form (LC3-II). Therefore, the ratio of LC3-II/I can serve as the primary protein of autophagy [22]. The scaffolding adaptor p62 can connect the LC3 protein to an ubiquitination substrate and can be incorporated into the complete phagosome by final autophagic lysosomal to degradation, marking the completion of autophagic flux [23]. Autophagic flux included the fusion autophagosomes with lysosomes and subsequent degradation of cargo, which was assessed using western blotting to detect the protein expression of LC3 II/I and p62 with and without lysosomal protease inhibitors to block autophagic degradation [24]. In our study, the results of western blotting indicated that AGEs could simultaneously increase the ratio of LC3-II/I as well as p62 in HUVECs (Figure 3A). To better assess the influence of AGEs on autophagy and autophagic flux, we used chloroquine (CQ), an inhibitor of autophagosome degradation, to investigate whether the increased LC3-II/I was because of the induction of early autophagy or because of the inhibition of autophagic degradation. Cells were treated with AGEs (100 µg/mL) and/or CQ (10 µmol/L) for 24 h. As shown in Figure 3A–C, CQ could simultaneously increase the ratio of LC3-II/I and the protein expression of p62 in BSA control group and AGEs-treated group. Consistently, immunofluorescence assay illustrated that AGEs could increase the expression of p62, and CQ could further increase p62 expression in AGEs-treated group (Figure 3D,E). Acridine orange (AO) staining exhibited that AGEs could increase the accumulation of acidic vacuoles in HUVECs, and CQ could further increase the acidic vacuoles in AGEs-treated group (Figure 3F). Overall, these results indicated that AGEs increased the autophagic flux in HUVECs, in accordance with previous studies [25,26]. When autophagic flux was blocked by CQ, the results of MTT assay (Figure 3G) and EdU staining (Figure 3H) indicated that cell viability and proliferation further decreased compared to AGEs-treated group.

### 2.4. Cathepsin D (CTSD) Was Implicated in Autophagic Flux Mediated by AGEs in HUVECs

**Figure 4:**
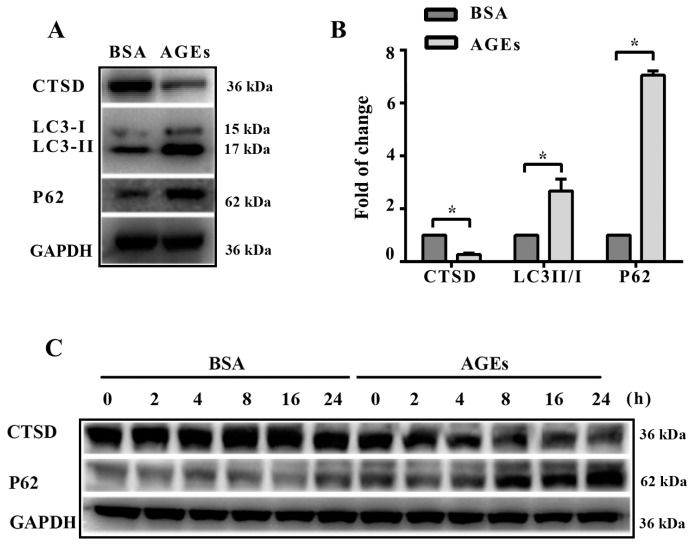

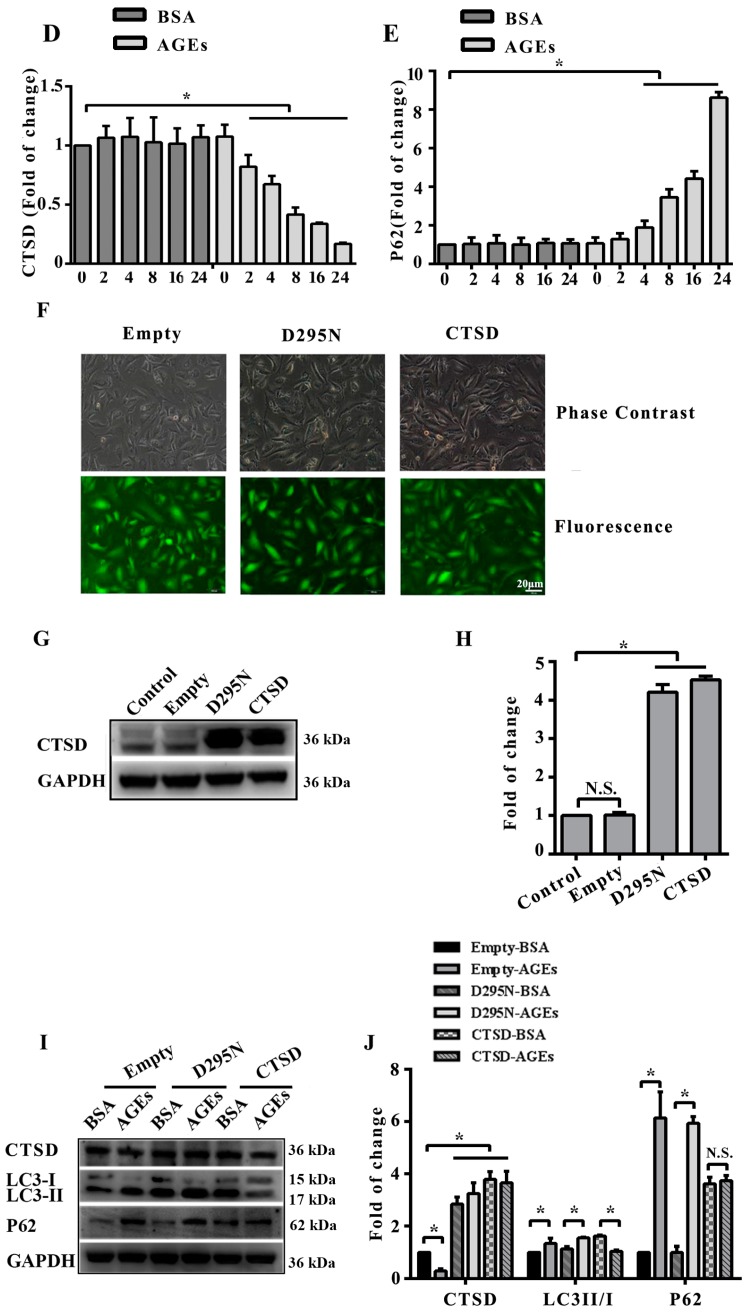
Cathepsin D (CTSD) was implicated in autophagic flux mediated by AGEs in HUVECs. CTSD was overexpressed in HUVECs using lentiviral vectors labelled with GFP. (**A**) Cells were treated with 100 µg/mL AGEs and 10 µg/mL BSA for 24 h. The protein expression of LC3-II, LC3-I, p62, and CTSD were analyzed using western blotting, and GAPDH was used as a control; (**B**) The band intensity was quantified using Image J 1.47, normalized by GAPDH (* *p* < 0.05 vs. BSA group); (**C**) Cells were treated with 100 µg/mL AGEs and 10 µg/mL BSA for different times (0, 2, 4, 8, 16 and 24 h). The expression of p62 and CTSD were analyzed using western blotting, and GAPDH was used as a control; (**D**,**E**) The band intensity was quantified using Image J 1.47, normalized by GAPDH (* *p* < 0.05 vs. BSA group at 0 h); (**F**) CTSD was successfully transfected into cells. Green cells were labelled with green fluorescent protein, which indicated the transfection was successful; (**G**) The protein expression of CTSD was analyzed by western blotting; (**H**) The band intensity was quantified using Image J 1.47, normalized by GAPDH (* *p* < 0.05 vs. normal control. N.S., no statistical significance.); (**I**) The protein expression of LC3-II, LC3-I, p62, and CTSD were analyzed using western blotting after CTSD was transfected into cells; (**J**) The band intensity was quantified using Image J 1.47, normalized by GAPDH (* *p* < 0.05 vs. empty-BSA group. N.S., no statistical significance.). All data were obtained from reproducible experiments (*n* = 3).

Lysosomes play a crucial role in autophagy, where misfolded proteins and cell debris in autophagic vacuoles could be degraded by lysosomal enzymes. We next aimed to assess whether autophagic flux was associated with lysosomal enzymes. The results of western blotting indicated that the expression of CTSD significantly decreased after treatment with AGEs (Figure 4A,B). Figure 4C–E shows that CTSD reduction coincided with p62 accumulation. CTSD participates directly in autophagy and is responsible for the degradation of autophagosomes [20]. Our results seemed to be contradictory to previous studies.

To explore the role of CTSD and its enzyme activity in autophagy induced by AGEs, CTSD was overexpressed in HUVECs using lentiviral vectors labelled with green fluorescent protein (GFP). The GFP-expressing lentiviral plasmids containing wild-type or catalytically inactive mutant (D295N) CTSD complementary deoxyribonucleic acid (cDNA) were transduced into cells, which can express wild-type CTSD protein (with enzyme activity) and mutant CTSD protein (without enzyme activity), respectively [27]. Measurement of GFP positive cells under fluorescence microscopy showed that lentiviral vectors were successfully transduced into cells with transduction efficiency >90% when multiplicity of infection (MOI) was 100 (Figure 4F). Western blotting also confirmed that the lentiviral CTSD construct could significantly increase CTSD expression (Figure 4G,H). Importantly, wild-type CTSD could significantly decrease the ratio of LC 3II/I as well as p62 accumulation induced by AGEs, but D295N CTSD had no such effects (Figure 4I,J), indicating that CTSD overexpression could further promote the degradation of autophagosome and that this effect was related with its enzymatic activity.

### 2.5. Overexpression of CTSD Recovered AGEs-Inhibited Proliferation of HUVECs

**Figure 5:**
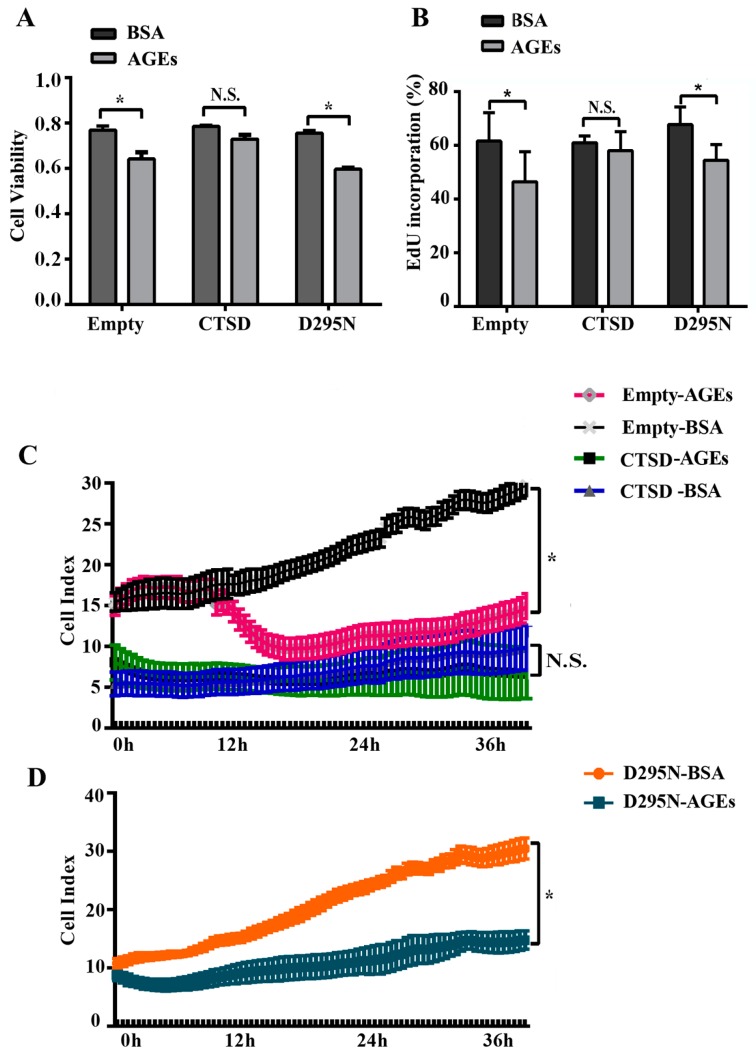
The effects of CTSD on the proliferation of HUVECs. Lentiviral plasmids containing wild-type CTSD or D295N CTSD complementary deoxyribonucleic acid (cDNA) were transduced into cells, which were classified into the CTSD group and D295N group, respectively. Cells with empty vector served as control. (**A**) Cell viability was detected by MTT; (**B**) EdU incorporation was quantified using Image J 1.47; (**C**,**D**) Cell proliferation was monitored by RTCA dynamically. Cell index was recorded automatically at 15 min intervals and monitored continuously for 36 h. All data were obtained from reproducible experiments (*n* = 3). * *p* < 0.05 compared with respective BSA group. N.S., no statistical significance.

As previous study indicated, autophagy played an important role in regulating the proliferation of cells [21]. CTSD, an aspartyl protease in lysosomes, participates directly in autophagy [20]. Therefore, CTSD may be also implicated in AGEs-induced proliferation and migration of HUVECs. The results of MTT assay (Figure 5A), EdU incorporation assay (Figure 5B), and RTCA (Figure 5C,D) all showed that overexpression of CTSD, due to its catalytic activity, could recover the proliferation ability of HUVECs inhibited by AGEs.

### 2.6. Overexpression of CTSD Recovered AGEs-Inhibited Migration of HUVECs

**Figure 6:**
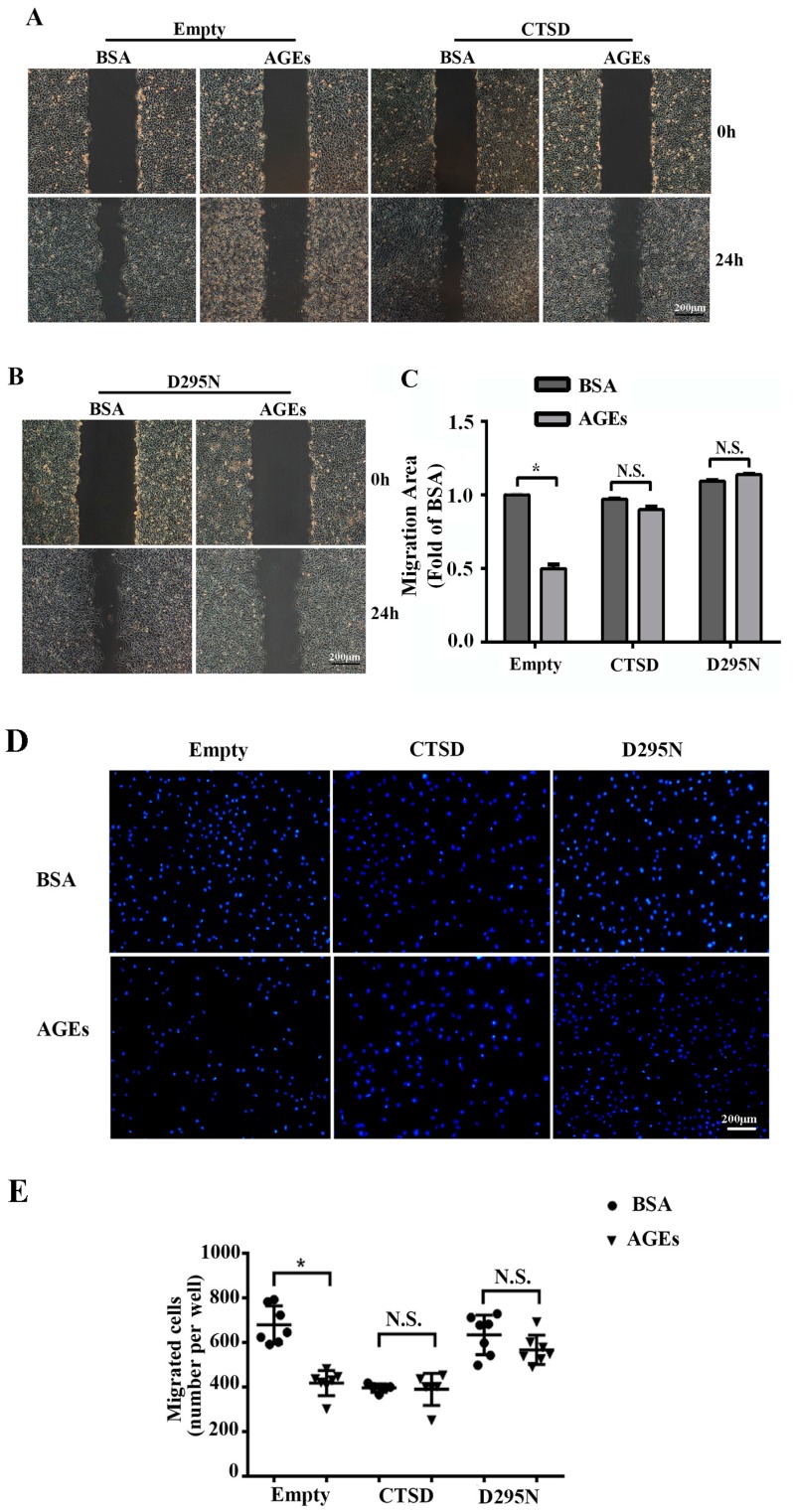
The effects of CTSD on the migration of HUVECs. Lentiviral plasmids containing wild-type CTSD or D295N CTSD cDNA were transduced into cells, which were classified into the CTSD group and D295N group, respectively. Cells with empty vector served as control. Cells were treated with 100 µg/mL AGEs or 10 µg/mL BSA as control, and incubated with serum-free DMEM for 24 h to inhibit cell proliferation. (**A**,**B**) Cell migration was analyzed by wound-healing assay; (**C**) The migration area was quantified by Image J 1.47, normalized by BSA group (* *p* < 0.05 vs. BSA + empty group. N.S., no statistical significance.). Three microscopic fields were randomly selected; (**D**) Cell migration was also analyzed by Hoechst 33342 staining in the transwell assay. Blue dots represented nuclei. Three microscopic fields were randomly selected; (**E**) Migrating cells were counted under microscopy (* *p* < 0.05 vs. BSA + empty group. N.S., no statistical significance.). All data were obtained from reproducible experiments (*n* = 3).

To explore whether overexpression of CTSD could recover AGEs-inhibited migration of HUVECs, wound-healing assay and transwell migration assay were conducted. We found that overexpression of CTSD could improve the migration of AGEs-treated HUVECs regardless of its enzymatic activity (Figure 6A–E).

## 3. Discussion

In this study, we investigated the effects of AGEs on the proliferation and migration in HUVECs and explored its underlying molecular mechanism. Our results showed that AGEs exerted an inhibitory effect on the proliferation and migration and promoted autophagic flux in HUVECs. Autophagy played a protective role against AGEs-induced cell injury. Overexpression of CTSD could recover AGEs-inhibited proliferation and migration of HUVECs.

The effects of AGEs on endothelial cells have been studied for several decades. However, whether AGEs can promote or inhibit the proliferation and migration of HUVECs is still matter of debate [12,13,14,15]. Tezuka et al. [15] reported that AGEs did not affect the proliferation of HUVECs at a low concentration (5 µg/mL) but exerted an inhibitory effect at a high concentration (50 µg/mL). However, AGEs could markedly increase the migration of HUVECs in a concentration-dependent manner and the maximal activity was observed at a concentration of 5 µg/mL [15]. Wang et al. [14] reported that AGEs could markedly increase the proliferation and migration of HUVECs at three concentrations (50, 100 and 200 µg/mL) by inducing moesin phosphorylation via Ras homolog gene family, member A (RhoA)/RhoA-associated protein kinase (ROCK) pathway. However, in another study [12], late endothelial progenitor cells (EPCs) were treated with four concentrations (50, 100, 200 and 500 µg/mL) of AGEs and researchers found that AGEs could inhibit late EPC migration in a concentration-dependent manner. The inhibitory effect of AGEs on migration was related to AGEs-induced decrease in the stromal cell derived factor-1(SDF-1)/C-X-C motif chemokine receptor 4 (CXCR4) system. Though EPCs are different from HUVECs, they could express a variety of endothelial markers and functionally differentiate into mature endothelial cells [12]. Consistently, Chen et al. [28] also demonstrated that AGEs (50, 100, and 200 µg/mL) could inhibit the proliferation, migration, and adhesion, and induce apoptosis in EPCs through RAGE-mediated oxidant stress. In agreement with those findings, our results indicated that AGEs (50, 100, 150 and 200 µg/mL) could inhibit the proliferation and migration in HUVECs. The possible explanations for the differences in the observed relationship between AGEs and endothelial cell functions could be that: (1) the different types of endothelial cells may respond to AGEs variably. Primary HUVECs [14,15], late EPCs [12,28], and the HUVEC line (in our study) were used in different studies; (2) the different concentrations of AGEs (5, 10, 50, 100, 150, 200, 500 µg/mL) varied in different studies; (3) the methods for preparing AGEs varied in different studies. Because AGEs are a heterogeneous group of complex compounds via a series of non-enzymatic chemical reactions [14], the different incubation conditions may generate different active compounds in AGEs.

An increasing studies have demonstrated that autophagic–lysosomal pathway played a crucial role in removing dysfunctional cytoplasmic proteins and cellular organelles [3,4,16,17]. Previous studies indicated that autophagy could reduce cell injury and proliferation in HUVECs induced by glycated collagen I, an important component of AGEs [19]. Xie et al. [3] reported that AGEs could injure cells and at the same time activate autophagy in HUVECs via reactive oxygen species (ROS), while in turn autophagy could protect cells against AGEs-caused injury. On one hand, autophagy can remove damaged organelles, misfolded proteins and cell debris [3,4,16,17]; on the other hand, autophagy can also promote survival by generating the free amino acids and fatty acids which are required to maintain function during nutrient-limited conditions [3,4]. Our study indicated that autophagic flux in HUVECs was promoted with exposure to AGEs for 24 h. The ratio of LC3-II/I can serve as the primary protein of autophagy [22]. P62 is a marker of the completion of autophagic flux [23]. With the development of autophagy research, it is widely accepted that an increase in the number of autophagosomes alone does not necessarily correlate with increased autophagic activity or flux [3]. Although previous studies [3,19] demonstrated that AGEs and glycated collagen I (an important component of AGEs) could activate autophagy in HUVECs, only LC3-II/I was detected to state autophagosome formation. In one study, Xie et al. [3] treated the cells with AGEs for 6 h and only detected LC3II, which could not elucidate the change of autophagic flux. Therefore, we detected both LC3-II/I and p62 to access the autophagic flux. Our results showed that AGEs could increase both the ratio of LC3-II/I and the expression of p62, while AGEs plus CQ could further increase LC3-II and p62 levels (as compared to that of AGEs alone). These results indicated that AGE increased the autophagic flux [25,26]. When autophagic flux was blocked by CQ, the cell viability and proliferation further decreased compared to AGEs-treated group, which indicated that autophagy activation played a protective role in AGEs-induced cell injury. Our results were consistent with a previous study [3].

When autophagolysosomes form, lysosomal proteases participate directly in the process of autophagy and can degrade the contents in autophagosome [20]. CTSD, an aspartyl protease in lysosomes, is responsible for the degradation of cellular structures [20,21]. Our results indicated that AGEs could markedly decrease the expression of CTSD. How do AGEs downregulate CTSD, which is mainly located in the lysosomes? Microarray analysis from a previous study found that many messenger ribonucleic acids (mRNAs) were dramatically down-regulated in human retinal pigment epithelial cell when exposed to AGEs [29]. A large number of the down-regulated mRNAs could encode for lysosomal and degradative enzymes including the cathepsins (such as cathepsin D) [29]. It was further confirmed in their following verification experiments that AGEs could significantly reduce CTSD enzymatic activity in ARPE-19 [29]. As a previous study indicated, lysosomal enzymes were susceptible to age-related changes [30]. Dysfunction of CTSD was proven to be significantly correlated with accumulated autofluorescent inclusion in the cells exposed to AGEs [29]. Therefore, we speculated that AGEs downregulated CTSD via the RAGE–ROS pathway [3]. It is worth noting that CQ can be also seen as an inhibitor of CTSD because CQ can increase the pH value of lysosomes. The acidic pH in lysosomes can foster the most active and stable conformation of CTSD and other proteases [31]. As we all know, CTSD is an important protease but not the only one in the degradation of autophagosome. Cathepsins consist of a variety of members, including cysteine cathepsins (such as cathepsin B, cathepsin H, cathepsin L, and cathepsin S), aspartic acid cathepsin (i.e., CTSD), and serine cathepsins (cathepsin A and cathepsin G) [32]. Previous studies have indicated that cathepsin B [33] and cathepsin S [34] also play an important role in driving proteolytic degradation within the lysosomes. Why would overexpression of CTSD contribute to the recovery of AGEs-induced injury when CTSD could be compensated by other proteases in the autophagy-lysosome pathway? A previous study demonstrated that CTSD could degrade AGEs-modified albumins such as AGEs-BSA, however cathepsin B was less effective, and the 20S proteasome was completely unable to degrade AGEs-modified albumins such as AGEs-BSA [31]. Besides binding with its receptor RAGE, AGEs could also enter the cells via early/late endosomes and be directly delivered to lysosomes [35], where AGEs can only be degraded by CTSD [31]. Oxidized proteins are often a better substrate than nonoxidized ones, but the AGEs seem to maintain almost the same proteolytic susceptibility after BSA is modified [31]. At the same time, lysosomal enzymes are susceptible to age-related changes such as the accumulation of AGEs [30]. There is a balance between AGEs and CTSD. On one hand, CTSD is almost the only protease which is directly delivered to lysosomes by endocytosis to degrade AGEs; on the other hand, AGEs can decrease the expression of CTSD. Taken together, CTSD, as well as other proteases, plays an important role in degradation of autolysosome in autophagy-lysosome pathway; at the same time, only CTSD can directly degrade AGEs which are directly delivered to lysosomes by endocytosis. That is how overexpression of CTSD recovers AGEs-inhibited proliferation and migration of HUVECs. Furthermore, wild-type CTSD could recover the proliferation of HUVECs inhibited by AGEs, while D295N-CTSD did not show such effect, indicating the catalytic activity of CTSD was necessary for this effect. Additionally, overexpression of CTSD could improve the migration of AGEs-treated HUVECs regardless of its enzymatic activity, indicating that other mechanisms might exist. The exact mechanisms are so complex that further studies are still necessary in the future.

**Figure A1:**
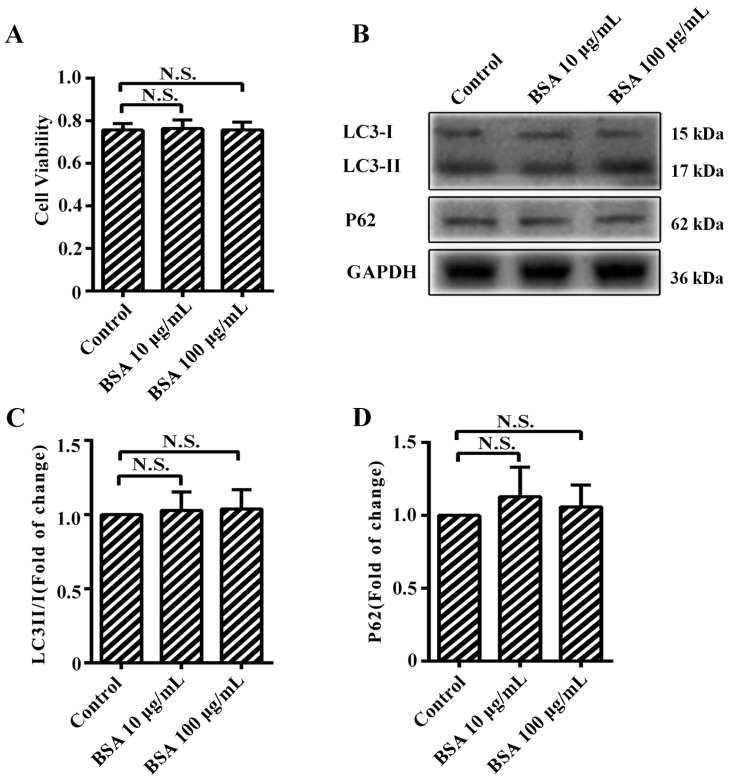
The effect of 100 µg/mL BSA on cell viability and autophagy. (**A**) Cell viability was detected by methyl thiazolyl tetrazolium (MTT) assay; (**B**) The protein expression of microtubule-associated protein 1 light chain 3 (LC3)-II, LC3-I, and p62 were analyzed using western blotting, and glyceraldehyde 3-phosphate dehydrogenase (GAPDH) was used as a control; (**C**,**D**) The band intensity was quantified using Image J 1.47, normalized by GAPDH. * *p* < 0.05 compared with control group. N.S., no statistical significance.

Our study has one limitation. Most studies of AGEs have used BSA at the same doses as that of AGEs [3,4,9,11]. Meanwhile, some studies also used a single dose of BSA as control with several doses of AGEs [12,14,28]. In our study, the dose of BSA was as low as 1/10 of that of AGEs, which was consistent with a previous study [21]. Generally speaking, BSA (like other controls) would not have an effect on cells, as shown in Figure 1B and Figure A1.

## 4. Materials and Methods

### 4.1. Materials

BSA and AGEs-BSA (AGEs, for short) were obtained from Merck-Millipore (Darmstadt, Germany). Fluorescence-activated cell sorter analysis was purchased from Beckman Coulter (Fullerton, CA, USA). xCELLigence Real-Time Cell Analyzer (RTCA) dual Plate (DP) was purchased from ACEA Biosciences (San Diego, CA, USA). A transwell (8-µm pores) was purchased from BD Biosciences (Franklin Lakes, NJ, USA). Fluorescence microscopy (CKX41-F32FL) was purchased from Olympus (Tokyo, Japan). Microchemi 4.2 was purchased from DNR (Jerusalem, Israel). Bicinchoninic acid (BCA) Protein Assay kit, Hoechst 33342 and 4′,6-diamidino-2-phenylindole (DAPI) were purchased from Beyotime (Shanghai, China). Reagents including acridine orange (AO), chloroquine (CQ), methyl thiazolyl tetrazolium (MTT), dimethylsulfoxide (DMSO), penicillin-streptomycin, and trypsin were purchased from Sigma-Aldrich (St. Louis, MO, USA). Primary antibodies including CTSD (mouse, product#: SAB1405677), p62 (rabbit, product#: SAB4504379), LC3 (product#: L8918) and glyceraldehyde 3-phosphate dehydrogenase (GAPDH, rabbit, product#: SAB2701826), and secondary antibody (mouse, product#: SAB1405848; rabbit, product#: SAB2100894) were purchased from Sigma (St. Louis, MO, USA). Click-iT™ EdU Alexa Fluor 555 Imaging Kit, NuPAGE Novex 4%–12% Bis-Tris Gel and XCell SureLock™ Mini-Cell were purchased from Invitrogen (Carlsbad, CA, USA). A microplate reader was purchased from Bio-Rad (Hercules, CA, USA). Enhanced chemiluminescence (ECL) was purchased from Amersham Biosciences (Piscataway, NJ, USA). Fetal bovine serum (FBS) and Dulbecco’s modified Eagle medium (DMEM) were purchased from Hyclone (Logan City, UT, USA).

### 4.2. Cell Culture

The HUVEC line was obtained from the Cell Bank of the Chinese Academy of Sciences (Shanghai, China). The cells were cultured at 37 °C in a humidified atmosphere with 5% CO_2_. Culture medium was DMEM supplemented with 10% FBS, 100 U/mL penicillin and 100 µg/mL streptomycin, which was replaced every 2 days or as necessary. In the experiments exploring the effect of AGEs on the proliferation and migration, HUVECs were incubated with 100 µg/mL AGEs or 10 µg/mL BSA as control for 24 h according to the MTT assay [21], as described in detail below. In the experiments exploring the effect of AGEs on autophagy, HUVECs were treated with 10 µM CQ (an autophagy inhibitor) for 30 min, and then exposed to 100 µg/mL AGEs or 10 µg/mL BSA for 24 h. Trypsin (0.25%) was used for digestion.

### 4.3. Overexpression of CTSD in HUVECs Using Lentiviral Vectors

As our previous study described [36], the GFP-expressing lentiviral plasmids containing wild-type or catalytically inactive mutant (D295N) CTSD cDNA were produced by GeneChem Corporation (Shanghai, China) and used in the CTSD-overexpression experiments. In brief, cells were seeded into a 6-well plate and incubated with vector supernatants for 12 h. Then, the old culture media was removed and replaced with DMEM with 10% FBS. After culture for 2 days, cells were observed under fluorescence microscopy and transduction efficiency was calculated using the following formula: transduction efficiency = GFP^+^ cells/all cells. After digestion, transduced cells were cultured for another 2 days. Cellular proteins were extracted and the expression of CTSD was detected using western blotting to see whether the transduction was successful.

### 4.4. Cell Viability Assays

Cell viability was analyzed using the MTT assay, as described in our previous study [37]. In brief, cells were seeded in a 96-well plate at a density of 2 × 10^3^ cells/well. The medium was removed after 24 h. Thereafter, cells were treated with various concentrations (50, 100, 150 or 200 µg/mL) of AGEs or various concentrations (5, 10, 15 or 20 µg/mL) of BSA as control for different times (12, 24, or 48 h). Cells were incubated with 0.5 mg/mL MTT for another 4 h in the dark at 37 °C before replacement with 100 µL DMSO. Then the plate was gently rotated on a linear and orbital shaker for 5 min to completely dissolve the precipitation. A microplate reader was used to determine the absorbance at 570 nm. Cell viability was calculated using the formula. Cell viability (%) = optical density (OD) of AGEs-treated group/OD of BSA-treated group × 100%.

### 4.5. Growth Curve Assays Using Real-Time Cell Analyzer (RTCA)

The RTCA system, as an advanced form of technology, can monitor cell growth real-timely using a label-free cell-based assay which can measure impedance variations in the culture media [38]. In the RTCA system, the cultured cells were attached to the cell culture wells which were incorporated in special cell culture plates, E-plates. Then, any changes in the electronic impedance were recorded through their sensors and expressed as the cell index, which was a parameter of cell viability [38]. To analyze the cell proliferation continuously over time, growth curve assays were performed in RTCA in quadruplicate with the xCelligence system according to the methods as previously described [39]. First, the E-plates were connected to the RTCA system to read background impedance, which ensures that all wells of E-plates and the connections were in good condition. Secondly, 5000 cells/well were seeded in the E-plates. After all chambers were set up, the RTCA E-plates were put into xCelligence instrument at 37 °C, 5% CO_2_ incubator and cell index was recorded automatically every 15 min intervals.

### 4.6. EdU Incorporation Analysis

As described by our previous studies [21,37], EdU incorporation assay was used to analyze DNA synthesis in HUVECs. Click-iT™ EdU Alexa Fluor 555 Imaging Kit was used according to the manufacturer’s instruction. Briefly, the cells were incubated with EdU-labeling solution for 8 h at 37 °C, and then fixed with 4% cold formaldehyde in the dark for 30 min at room temperature. Thereafter, cells were permeabilized with 1% Triton X-100 and incubated with Click-iT reaction cocktails for 30 min. Then, the Hoechst 33342 was used to stain the DNA contents for 30 min. In the end, EdU-labeled cells were counted using Image J 1.47. At least 500 cells in each experiment were counted and EdU incorporation was calculated using the formula. EdU incorporation (%) = EdU-positive cells (red dots)/(EdU-positive cells (red dots) + Hoechst-positive cells (blue dots)).

### 4.7. Scratch Wound-Healing Assay

Cells cultured in a 6-well plate were wounded by a 200 µL pipette tip to leave a gap between the two parts of cell monolayer. The cells were incubated in the serum-free medium plus respective stimulation in different groups (100 µg/mL AGEs or 10 µg/mL BSA) for 24 h. Cells were observed and photographed under the microscope at 0 and 24 h, respectively. The experiments were conducted three times with reproducible results. The migration areas were quantified using Image J 1.47 with the following steps. (1), Open image; (2), Image-Type-8 bit; (3), Process-Find edges; (4), Process-Sharpen; (5), Image-Adjust-Threshold; (6), Analyze-Analyze Particles.

### 4.8. Transwell Migration Assay

Transwell was set up in a 6-well plate, which was divided into two parts: the upper chamber and the lower chamber. The upper chamber contained DMEM without FBS while the lower chamber contained DMEM supplemented with 10% FBS plus 100 µg/mL AGEs or 10 µg/mL BSA as chemoattractant. Cells were seeded in the upper chamber at a density of 2 × 10^6^ cells. After culture for 24 h at 37 °C, a cotton bud was used to remove the cells on the upper chamber. Hoechst 33342 was used to stain the cells on the lower chamber, which represented migrating cells induced by AGEs or BSA. The experiments were conducted three times with reproducible results. The cells on the lower chamber were observed and photographed under fluorescence microscope with ultraviolet light. Hoechst-labeled cells in three representative microscopic fields were counted using Image J 1.47.

### 4.9. Western Blotting Analysis

Western blotting analysis was conducted according to our previous studies [37,40]. The total protein concentrations were determined using a BCA Protein Assay kit. After heat-denaturing, proteins were separated by NuPAGE Novex 4%–12% Bis-Tris Gel and electrophoresed in the XCell SureLock™ Mini-Cell. Thereafter, proteins were transferred onto a polyvinylidene fluoride (PVDF) membrane and blocked with 5% nonfat milk in Tris-buffered solution (TBS) for 1.5 h at the room temperature. The membranes were incubated with primary antibody overnight at 4 °C. Primary antibodies including CTSD, LC3, p62 and GAPDH were analyzed. All primary antibodies were used in a 1:1000 dilution in 1× Tris Buffered Saline with Tween-20 (TBS-T). After washing with 1× TBS-T (three times, 15 min/time), the PVDF membrane was incubated with secondary antibody in a 1:10,000 dilution for 1.5 h at room temperature. After washing with 1× TBS-T (three times, 15 min/time), immunoreactive bindings were detected with ECL. The band intensity was quantified with Image J 1.47 using the following steps. (1), Open image; (2), Edit-invert; (3), Image-Type-8 bit; (4), Analyze-calibrate-uncalibrated OD-OK; (5), select western blotting binding, analyze-gels-select first lane; (6), Analyze-gels-plot lanes; (7), select baseline and then click the peak area.

### 4.10. Acridine Orange (AO) Dyeing Assay for Autophagy Vesicles

AO could enter cell membrane and appear green under fluorescent microscopy. Meanwhile, AO could be protonated and trapped in acidic vesicular organelles such as autolysosomes and fluorescence red in a concentration-dependent manner. Taking the advantages of quickness and high reliability, AO staining was usually used to assess the volume of autolysosomes [41]. Cells were cultured at 37 °C in a 6-well plate with respective stimulation in different groups for 24 h. After rinsing with phosphate buffered saline (PBS), 1 mL of AO dyeing working solution (0.1 mg/mL, final concentration, in the dark) was added to the plate for 3 min. After rinsing with PBS, images were captured by fluorescent microscopy.

### 4.11. Immunofluorescence (IF) Staining and Fluorescence Microscopy

Paraformaldehyde (4%) was used to fix cells for 15 min at room temperature. After washing with PBS (three times, 15 min/time), 0.25% Triton X-100 was used to permeabilize cells for 5 min. After blocking with 1% BSA, cells were incubated with first antibody (1:200) overnight at 4 °C. After washing with PBS (three times, 15 min/time), cells were incubated with secondary antibody for 1 h in the dark. After washing with PBS (three times, 15 min/time), 0.1% DAPI was used to stain cell nucleus for 5 min. Finally, cells were observed and photographed under fluorescent microscopy. The fluorescence intensity was quantified with Image J 1.47 using the following steps. (1), Open image; (2), Image-type-32 bit; (3), Analyze-tools-region of interest (ROI) manager; (4), add the selected area into ROI; (5), Measure.

### 4.12. Statistical Analysis

All data were obtained from at least three independent experiments. Continuous variables were expressed as the mean ± standard deviation (SD) and tested by one-way ANOVA with Bonferroni post hoc analysis or Student’s *t*-test. Categorical variables were expressed as percentage and tested by chi-square test. All the statistical analyses were performed using SPSS 17.0 (Chicago, IL, USA: SPSS). *p* < 0.05 was considered to be statistically significant.

## 5. Conclusions

In summary, our study found that AGEs promoted an inhibitory behavior with respect to the proliferation and migration in HUVECs. AGEs promoted autophagic flux, which in turn played a protective role against AGEs-induced cell injury. CTSD, due to its catalytic activity, may function with a pro-proliferation role in the AGEs-treated HUVECs-independent autophagy-lysosome pathway. Meanwhile, CTSD could improve the migration of AGEs-treated HUVECs regardless of its enzymatic activity, which may refer to other mechanisms. These results may enhance understanding of AGEs-related autophagy in HUVECs and provide potential therapeutic targets for endothelial injury in diabetic angiopathies.

## Figures and Tables

**Figure A2:**
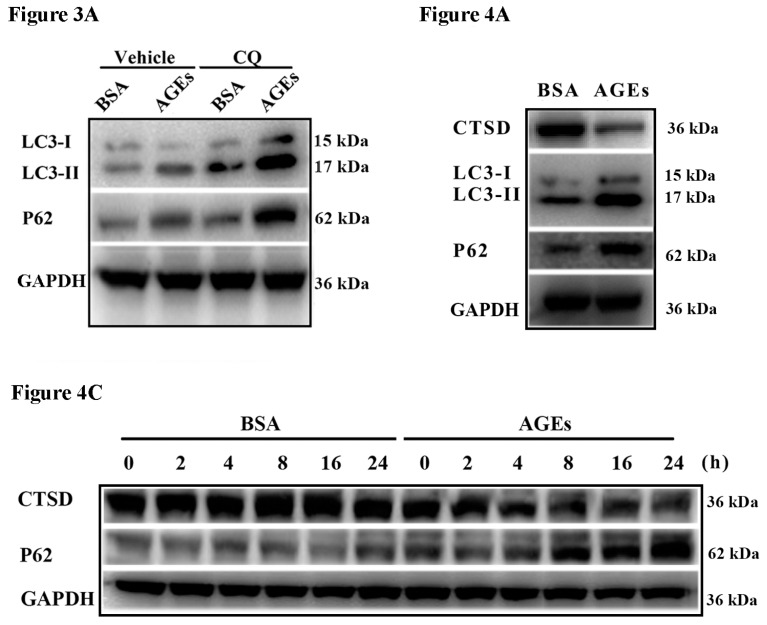

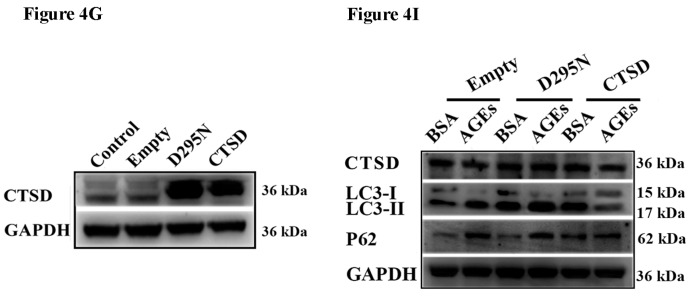
All the uncut western blotting bands in Figure 3 and Figure 4. AGEs: advanced glycation end products; CTSD: cathepsin D.

## Abbreviations

AGEs	Advanced glycation end products
AO	Acridine orange
cDNA	Complementary deoxyribonucleic acid
cGMP	Cyclic guanosine monophosphate
CQ	Chloroquine
CTSD	Cathepsin D
CXCR4	C-X-C motif chemokine receptor 4
DAPI	4′,6-Diamidino-2-phenylindole
DMEM	Dulbecco’s modified eagle medium
DMSO	Dimethyl sulfoxide
ECL	Enhanced chemiluminescence
EdU	5-Ethynyl-2′-deoxyuridine
EPCs	Endothelial progenitor cells
FBS	Fetal bovine serum
GAPDH	Glyceraldehyde 3-phosphate dehydrogenase
GFP	Green fluorescent protein
HUVECs	Human umbilical vein endothelial cells
LC3	Microtubule-associated protein 1 light chain 3
MOI	Multiplicity of infection
mRNA	Messenger ribonucleic acid
NF-κB	Nuclear factor-κB
NO	Nitric oxide
N.S.	No statistical significance
ox-LDL	Oxidized low density lipoprotein
PBS	Phosphate buffered saline
PVDF	Polyvinylidene fluoride
RAGE	Receptor of advanced glycation end products
RhoA	Ras homolog gene family, member A
ROCK	RhoA-associated protein kinase
RTCA	Real-time cell analyzer
ROI	Region of interest
ROS	Reactive oxygen species
SDF-1	Stromal cell derived factor-1
SD	Standard deviation
TBS	Tris-buffered solution
TBS-T	Tris-buffered saline with Tween-20
VSMCs	Vascular smooth muscle cells

## Appendix A

These experiments were conducted to illustrate that bovine serum albumin (BSA) did not have effect on cell viability and autophagy, as shown in Figure A1.

Figure A2 shows all the uncut western blotting bands in Figure 3 and Figure 4.
